# Neck shrivel in European plum is caused by cuticular microcracks, resulting from rapid lateral expansion of the neck late in development

**DOI:** 10.1007/s00425-023-04218-9

**Published:** 2023-08-05

**Authors:** Bishnu P. Khanal, Anil Bhattarai, Divya Aryal, Moritz Knoche

**Affiliations:** Institute for Horticultural Production Systems, Leibniz University Hannover, Herrenhäuser Straße 2, 30419 Hannover, Germany

**Keywords:** Allometry, Allometric growth, Cuticle, Fracture, *Prunus domestica*, Transpiration

## Abstract

**Main conclusion:**

Susceptibility to neck shrivel in European plum is due to cuticular microcracking resulting from high surface area growth rates in the neck region, late in development.

**Abstract:**

Susceptibility to the commercially important fruit disorder ‘neck shrivel’ differs among European plum cultivars. Radial cuticular microcracking occurs in the neck regions of susceptible cultivars, but not in non-susceptible ones, so would seem to be causal. However, the reason for the microcracking is unknown. The objective was to identify potential relationships between fruit growth pattern and microcracking incidence in the neck (proximal) and stylar (distal) ends of selected shrivel-susceptible and non-susceptible cultivars. Growth analysis revealed two allometric categories: The first category, the ‘narrow-neck’ cultivars, showed hypoallometric growth in the neck region (i.e., slower growth than in the region of maximum diameter) during early development (stages I + II). Later (during stage III) the neck region was ‘filled out’ by hyperallometric growth (i.e., faster than in the region of maximum diameter). The second category, the ‘broad-neck’ cultivars, had more symmetrical, allometric growth (all regions grew equally fast) throughout development. The narrow-neck cultivars exhibited extensive radial cuticular microcracking in the neck region, but little microcracking in the stylar region. In contrast, the broad-neck cultivars exhibited little microcracking overall, with no difference between the neck and stylar regions. Across all cultivars, a positive relationship was obtained for the level of microcracking in the neck region and the difference in allometric growth ratios between stage III and stages I + II. There were no similar relationships for the stylar region. The results demonstrate that accelerated stage III neck growth in the narrow-neck plum cultivars is associated with more microcracking and thus with more shrivel.

**Supplementary Information:**

The online version contains supplementary material available at 10.1007/s00425-023-04218-9.

## Introduction

Neck shrivel in plum is an important fruit surface disorder (Fig. S1) (Knoche et al. 2019). Fruit exhibiting shrivel symptoms is perceived as of low quality and is often excluded from the market. Susceptibility to neck shrivel varies between cultivars, indicating that genotype is involved, and it also varies between seasons, indicating that environmental factors play a role.

The mechanistic basis of neck shrivel is not clear. Earlier studies indicate that microcracks in the cuticle are causal. Microcracks occur at high frequency in the neck region of susceptible cultivars but not at all, or only at a low frequency, in the stylar scar region of these cultivars (Knoche et al. 2019). Microcracks are minute fissures in the cuticle that do not extend depthwise into the epidermis or hypodermis. Such microcracks impair the barrier functions of the cuticle, causing increased transpiration in susceptible fruit in the neck region (Knoche et al. 2019). At the same time, the water inflow to the fruit via the xylem decreases and almost ceases towards fruit maturity (Khanal et al. 2021). Thus, the water balance of the fruit becomes particularly negative in the stylar end region (Knoche and Grimm 2022) resulting in shrinkage and the well-known phenomenon of neck shrivel.

The reason for the development of radial microcracks on the fruit surface is unknown. It is well established in a number of fruit crops that microcracks result from growth strains (e.g., in sweet cherry, Peschel and Knoche 2005; in Ribes berries, Khanal et al. 2011 and in grapes, Becker and Knoche 2012). Furthermore, microcracking is exacerbated by surface moisture (Knoche and Peschel 2006). A priori, we would not expect plums to behave much differently. Indeed, earlier studies established that the cuticle of plum is significantly strained during the later phases of fruit development (Knoche and Peschel 2007). The strain results from a cessation of cutin deposition during late fruit development (final swell, stage III of stone fruit development) while wax deposition continues (Knoche and Peschel 2007). It is in about the pit-hardening phase (stage II of stone fruit development) when cutin deposition slows. Thus, in stage III an approximately constant amount of cutin must be distributed over an increasing area of fruit surface. This results in significant cuticular strain. Also, surface wetness increases microcracking in plum which is consistent with growers’ anecdotal observations with increases in the incidence of *Monilinia* fruit rots in wetter growing seasons. What is not known and is particularly difficult to explain is (i) why microcracking is limited to the (proximal) neck end of the fruit while the (distal) stylar end of the seemingly symmetrical fruit remains asymptomatic and (ii) why the alignment of the microcracks is highly oriented radially towards the pedicel (see Knoche et al. 2019). A potential explanation for the occurrence of radially oriented microcracks in the neck region would be a transverse expansion of the fruit surface in the neck region and a corresponding lack of such a transverse expansion in the stylar region.

The objective of this study was to test the hypothesis that susceptibility to neck shrivel is related to different patterns of surface growth in the neck and stylar regions. This hypothesis predicts that high susceptibility cultivars would exhibit particularly high rates of lateral expansion in the neck region and significantly less so in the stylar region.

## Materials and methods

### Plant materials

Fruits of European plum (*Prunus domestica* L.) cultivars Hauszwetsche Wolff and Cacaks Schöne were obtained from the experimental orchards of the Horticultural Research Station of the Leibniz University Hannover at Ruthe (lat. 52° 14ʹ N, long. 9° 49ʹ E) and also from the orchards of the Federal Fruit Variety Office at Wurzen, Germany. Fruits of all other cultivars were obtained only from the orchards of the Federal Fruit Variety Office at Wurzen. ‘Hauszwetsche Wolff’ is considered susceptible to neck shrivel, whereas ‘Cacaks Schöne’ is considered not susceptible.

### Monitoring fruit growth

Fruit were sampled and brought to the laboratory. Calibrated images were taken using a digital camera (Canon EOS 550D, EF-S 18–55 mm; Canon, Tokyo, Japan) mounted on a camera stand. Fruit length and fruit diameter were measured in the images using image analysis software (Cell^P^; Olympus Europa, Hamburg, Germany). The fruit surface area (${A}_{fruit}$) was calculated from the polar radius (*a*; fruit length divided by two) and the equatorial radius (*b*; fruit diameter divided by two) assuming the shape of the fruit as prolate ellipsoid (Knoche and Peschel 2007). The following equation was used

$${A}_{fruit}=2\pi b\left[\left(b+\frac{{a}^{2}}{\sqrt{{a}^{2}-{b}^{2}}}\right).\frac{arsin\left(\sqrt{{a}^{2}-{b}^{2}}\right)}{a}\right]$$

### Allometric growth of different regions of the fruit

Fruit of selected plum cultivars were sampled at two-week intervals between the middle of stage I development and maturity. Calibrated images of the fruit (cheek facing the lens) were prepared using a digital camera (Canon EOS 550D, EF-S 18–55 mm; Canon) mounted on a camera stand. Using image analysis software (CellP), the diameters of the fruit at seven different positions along the long axis were measured. The virtual transverse lines at those seven positions divide the fruit into eight virtual slices of equal thickness; the spacings between the virtual lines were measured, each of these being 1/8 (12.5%) of the fruit length. Subsequently, the log10 of all diameters and total length were calculated and plotted against the log10 of the center diameter. The slope of this relationship represents the rate of growth of each particular slice of the fruit relative to the growth in the center (allometric growth). Using the nomenclature suggested by Huxley (1924) this slope term is referred to as the ‘constant differential growth ratio’. A constant differential growth ratio of 1 means growth is allometric (i.e., fruit growth in some area of interest is equally rapid as in the center region). If the constant differential growth ratio is > 1 then growth is referred to as hyperallometric (i.e., fruit growth in some area of interest is more rapid than in the center region), correspondingly, if the constant differential growth ratio is < 1, then growth is hypoallometric (i.e., slower than in the center region). The constant differential growth ratios of the necks and the stylar regions of all selected plum cultivars were quantified.

Of the genotypes with sufficient fruit set, 15 cultivars were selected that fell into one or another of two, contrasting growth categories: the ‘narrow-neck cultivars’ (*n* = 7) all had a ratio of fruit diameters at 12.5% length to 50% length ≤ 0.7. For this group of narrow-neck cultivars the constant differential growth ratios for the neck region were ≤ 0.8 (Table S1). The ‘broad neck cultivars’ (*n* = 8) all had a ratio of fruit diameters at 12.5% length to 50% length > 0.7. Cultivars in this category had a constant differential growth ratio for the neck region > 0.8 (Table S1).

### Monitoring microcracking

Cuticular microcracking was analyzed using the procedure of Peschel and Knoche (2005). Briefly, whole fruit were dipped for 10 min in 0.1% (w/w) aqueous acridine orange (AO) (Carl Roth, Karlsruhe, Germany). Fruit were then removed, rinsed with deionized water and observed under a fluorescence binocular microscope (MZ10F, Leica Microsystems, Wetzlar, Germany). Calibrated images from neck, equatorial and stylar regions of the fruit surface were taken under incident fluorescent light (GFP-plus filter, 480–440 nm excitation, ≥ 510 nm emission wavelength; Camera DP71, Olympus).

All images were analyzed using Cell^P^ and the proportion of the fruit surface area infiltrated with AO was recorded (i.e., the relative area occupied by the microcracks) and the number of microcracks per unit area was counted. Acridine orange is known to penetrate through holes and openings in the fruit skin but to be excluded from penetration along the cuticular pathway (Middelberg et al. 2014).

It was found impossible to adequately quantify the lengths of the large number of microcracks in the neck region of the fruit either automatically or manually. Thus, the following procedure was developed. A total of 35 representative images from all three regions of the fruit of the various cultivars were selected, that spanned the full range of microcracking incidence. The areas infiltrated by AO, the length and width of the microcracks and the numbers of microcracks were quantified. A mean area per microcrack was calculated by dividing the total AO infiltrated area by the number of microcracks. The mean length of microcracks (‘calculated length’) was obtained by dividing the area per microcrack by the mean width of a microcrack. The calculated lengths and the measured lengths of the microcracks were highly correlated (*r* = 0.90***; Fig. S2a). This relationship shows that the length of the microcracks can be reliably estimated from the area per microcrack using the following equation:

$$ {\text{Mean length of microcracks }}\left( {{\text{mm}}} \right)\, = \,{5}.{49}\, \times \,{\text{area per microcrack }}\left( {{\text{mm}}^{{2}} } \right),\;r^{{2}} \, = \,0.{89}*** $$

This equation was used to calculate the average length of the microcracks in all the images of all the cultivars (Fig. S2b).

### Relationships between macrocracks and microcracks in the neck region

On some fruit, radial grey lines were visible to the naked eye. These represented macroscopic cracks (‘macrocracks’) and were always limited to the neck end of the fruit and always ran towards the pedicel. To identify whether these originated from cuticular microcracks, the relationship between the macrocracks and microcracks was analyzed.

Calibrated images of the neck region of the fruit were taken from above using binocular microscope (MZ6; Leica Microsystems) under incident bright light. In those images the number of macrocracks around the circumference of the neck region was counted. For counting two virtual lines, first at P-12.5 (i.e., 1/8 or 12.5% of the way along the fruit from the proximal end) and the second at P-6.25 (i.e., 1/16 or 6.25% of the way along the fruit from the proximal end) positions were drawn. Every crack that intersected the P-12.5 or the P-6.25 line was counted and the numbers averaged to yield the number of macrocracks per fruit in the neck region.

Subsequently, the fruit were dipped in 0.1% aqueous AO solution for 10 min, rinsed with deionized water and observed under the fluorescence binocular microscope (MZ10F). Representative images of the surface of the neck region between P-12.5 and P-6.25 position of the fruit were prepared under incidence fluorescence light (five images per fruit from different positions around the circumference).

Microcracks were counted on those fluorescence micrographs. Two parallel horizontal lines were drawn – one in the upper and one in the lower portion of the image. The numbers of microcracks intersecting either line were counted and the numbers averaged. Subsequently, the ‘number of microcracks per fruit’ (intersecting a virtual line of circumference in the neck region) was calculated by multiplying the average number of microcracks per unit length by the neck circumference.

### Data analysis and presentation

Data are presented as means ± standard errors. Data were analyzed by analysis of variance using the statistical software package SAS (version 9.4; SAS Institute, Cary, NC, USA).

## Results

**Fig. 1:**
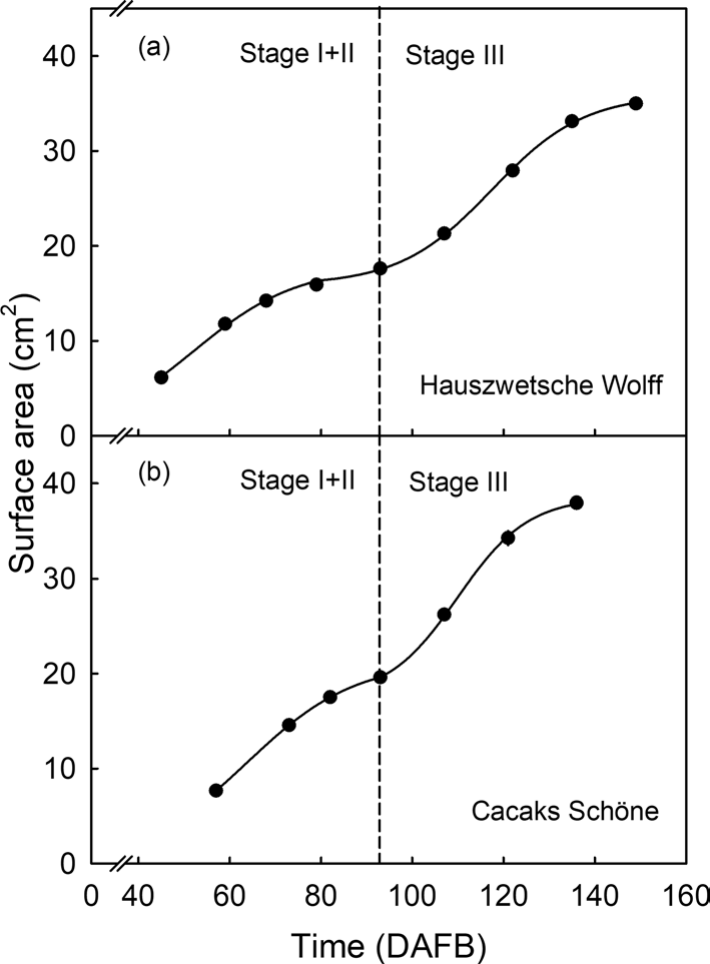
Time course of increase in surface area of the fruit of European plum cvs. ‘Hauszwetsche Wolff ‘(**a**) and ‘Cacaks Schöne’ (**b**)

The shrivel susceptible cv. ‘Hauszwetsche Wolff’ and the shrivel non-susceptible cv. ‘Cacaks Schöne’ both followed the same pattern of growth, typical of the later stages of development of most stone fruit species (Fig. 1). Typical stone fruit development is double sigmoid with stage I comprising the first sigmoid increase in mass, due primarily to cell division throughout the pericarp. Stage II is the phase of pit hardening and the onset of color change. The final stage III is characterized by a rapid increase in fruit mass, primarily due to cell expansion in the pericarp. The growth curve in Fig. 1 shows the stage II/III transition at about 93 days after full bloom (DAFB).

**Fig. 2:**
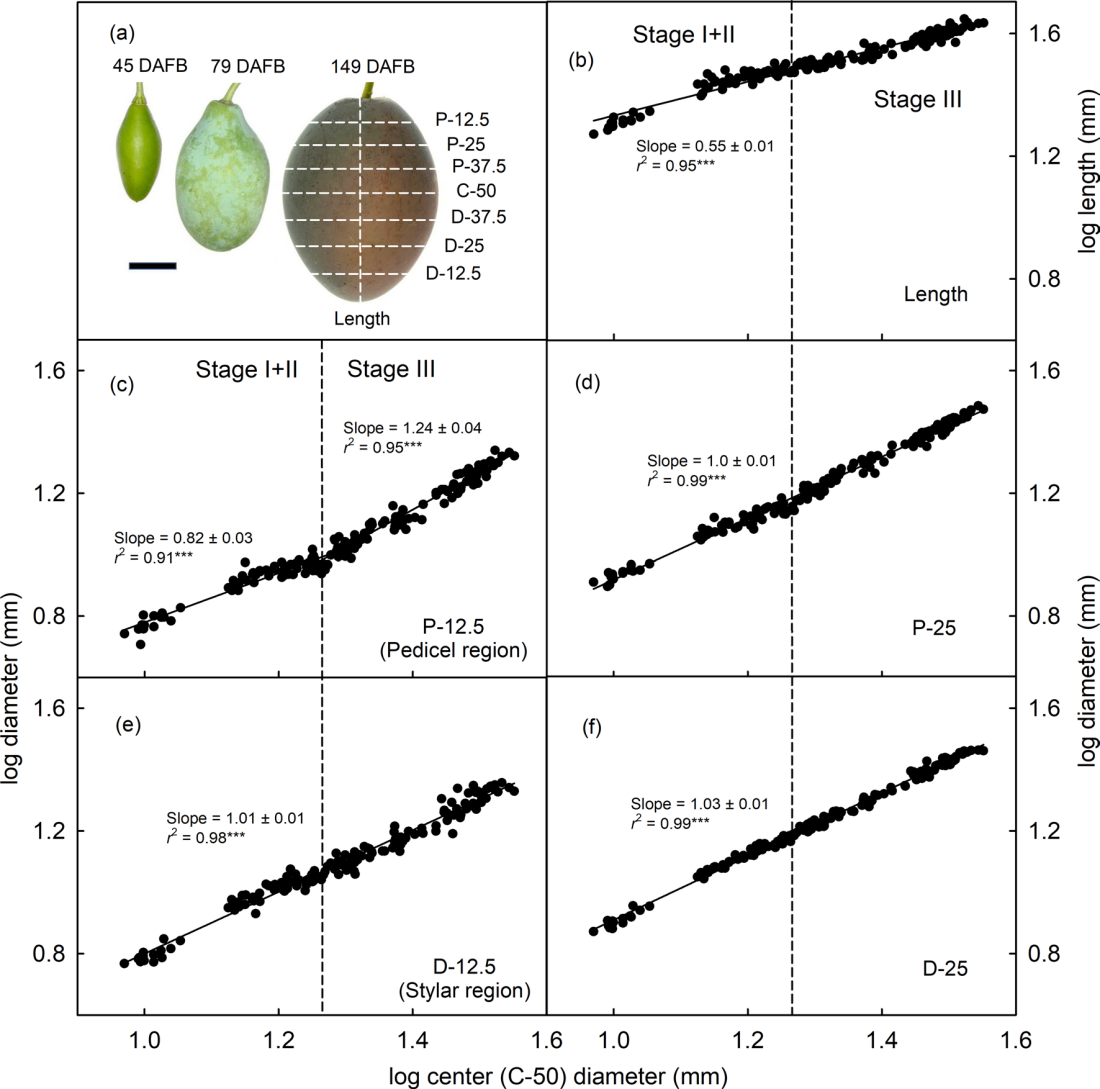
**a** Images of fruit of European plums cv. ‘Hauszwetsche Wolff’ at three different stages of development. **b–f** Relationship between the log transformed fruit lengths (**b**) or the log transformed fruit diameters of various regions (represented by horizontal dash lines in **a**) and log transformed fruit center diameter (**c–f**). Scale bar in **a** equals 10 mm. *DAFB* days after full bloom, *P* proximal, *C* center, *D* distal

The growth analysis for ‘Hauszwetsche Wolff’ indicates allometric growth in length and diameter in most regions as indexed by a constant slope of the log log relationships of growth in dimensions. The only exception was the growth in diameter of the most proximal part of the neck (P-12.5) of the fruit. The breakpoint indicated a biphasic growth pattern. Up to 93 DAFB (corresponding to the stage II/III transition) the growth in diameter in this region of the neck was hypoallometric (i.e., slower than the growth in the maximum diameter region). From the breakpoint onwards, the growth in this region of the neck accelerated and became hyperallometric (i.e., faster than the growth in the maximum diameter region) (Fig. 2). This pattern was consistent, with a change in shape of the proximal portion of the fruit between 45 and 149 DAFB. During this time, the proximal quarter of the fruit ‘filled out’ to yield a symmetrical fruit at maturity (Fig. 2).

**Fig. 3:**
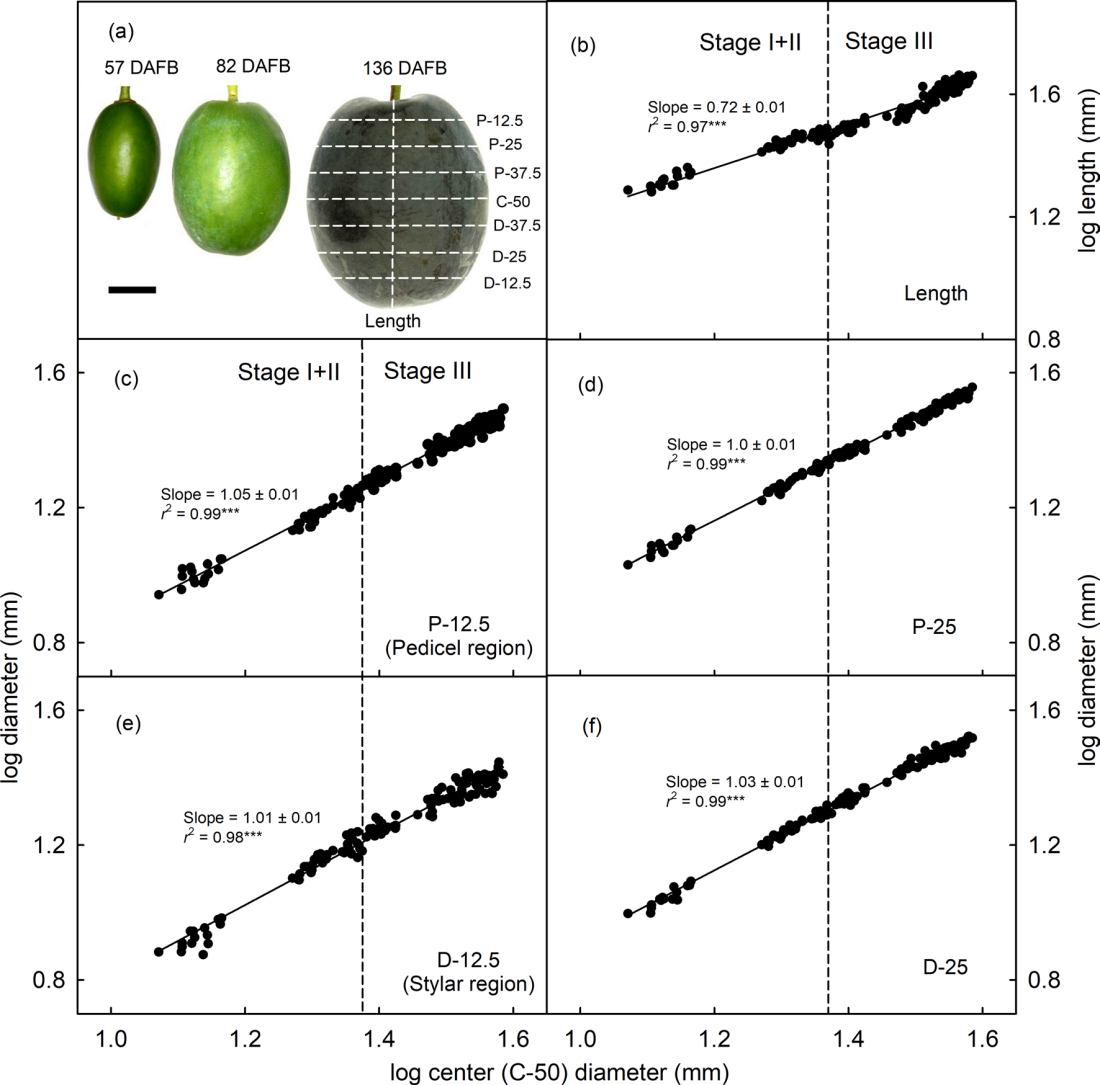
**a** Images of fruit of European plums cv. ‘Cacaks Schöne’ at three different stages of development. **b–f** Relationship between log transformed length (**b**) or log transformed diameter of the fruit in various regions (represented by the horizontal dashed lines in **a**) and log transformed center diameter of the fruit (**c–f**). Scale bar in **a** equals 10 mm. *DAFB* days after full bloom, *P* proximal, *C* center, *D* distal

When the same analyses were applied to developing cv. ‘Cacaks Schöne’, fruit growth was symmetrical throughout the development period. Linearity of the relationships of the log transformed diameters or length with log maximum diameter and a slope of about 1 indicates allometric growth in all dimensions (Fig. 3).

**Fig. 4:**
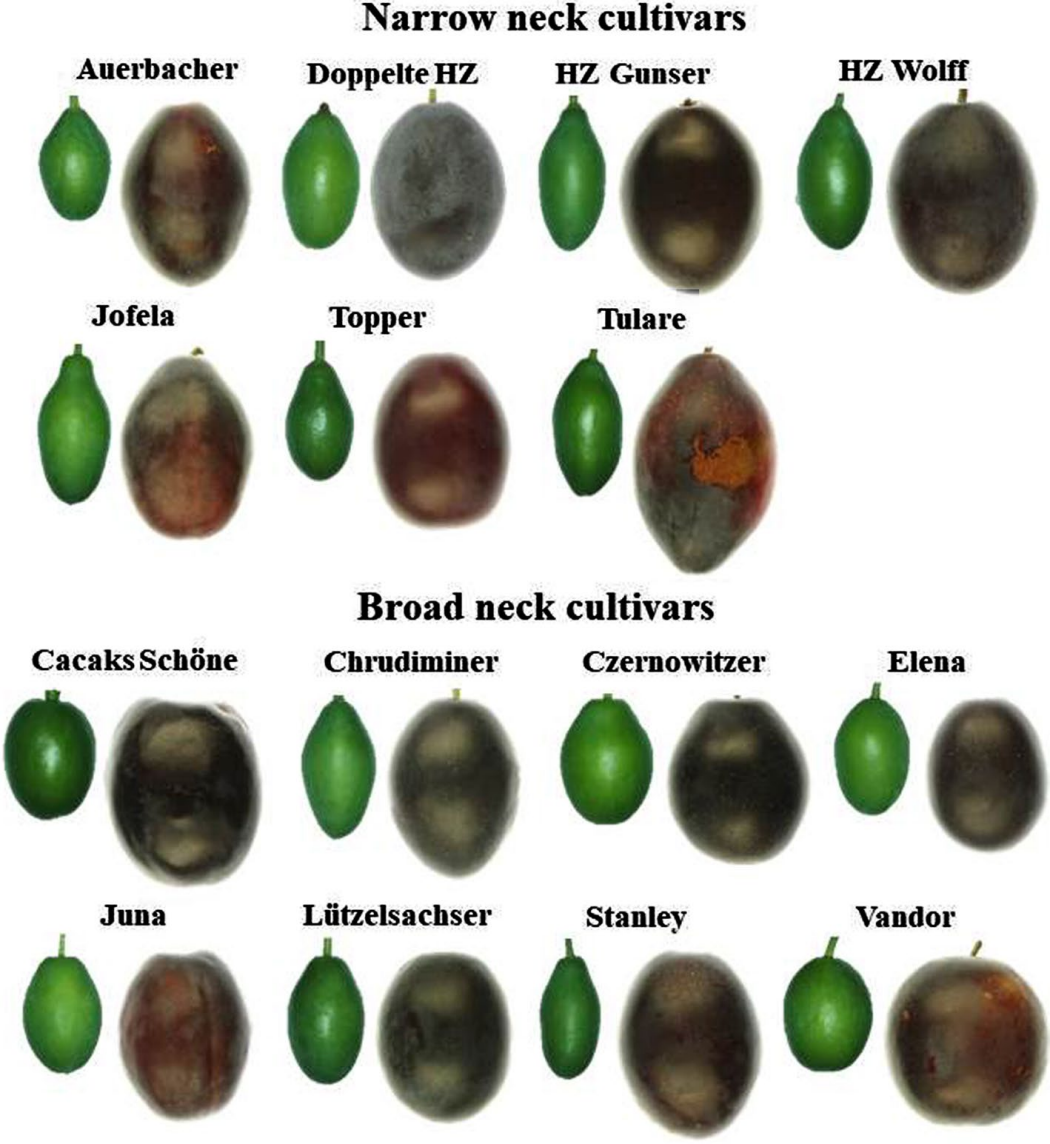
Images of developing fruit of European plums of various cultivars. The cultivars were grouped into two categories of ‘narrow-neck cultivars’ and ‘broad-neck cultivars’ based on the shape or fullness of the proximal (neck) region when young. *HZ* ‘Hauszwetsche’

Extending the analysis to a range of plum cultivars revealed two different categories of growth pattern. The fruit of cultivars of the first category had relatively narrow necks during early growth, stages I + II, but the narrow neck disappeared during stage III growth towards maturity. Meanwhile, the growth of the fruit of cultivars of the second category was approximately symmetrical throughout development. We refer to these two contrasting groups of cultivars as the ‘narrow-neck cultivars’ and the ‘broad-neck cultivars’. For representative images see Fig. 4.

**Fig. 5:**
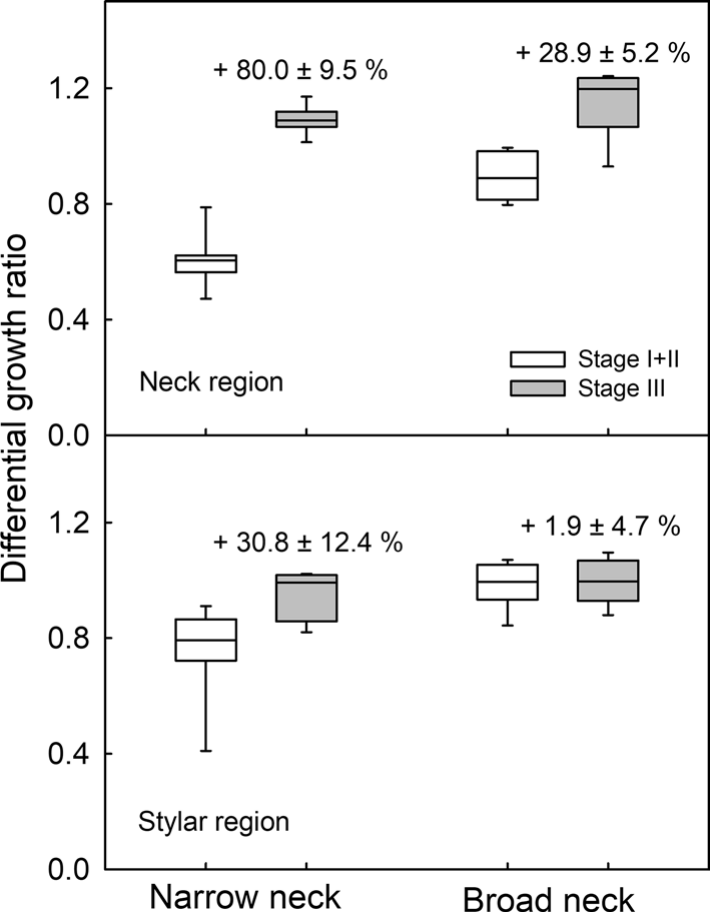
Boxplot of the constant differential growth ratios obtained as the slopes of the relationships between log transformed diameter of the proximal (neck; P-12.5) region or distal (stylar; D-12.5) region and the log transformed maximum diameter of the center region of fruit of selected European plum cultivars. Slopes were determined separately for stage I + II and for stage III fruit (as indicated in Figs. 2 and 3). The plum cultivars selected were either narrow-neck or broad-neck cultivars. The numbers in the Figures represent the relative increases in differential growth ratio from stage I + II to stage III

The allometric analyses reveal the fruit growth patterns are qualitatively and quantitatively different between the shrivel-susceptible and non-susceptible cultivars. This is shown in Figs. 2 and 3. In the narrow-neck cultivars, there was a marked increase (80.0 ± 9.5%) in the differential growth ratio between stage I + II and stage III in the neck region, while in the stylar region the increase was much smaller (28.9 ± 5.2%) (Fig. 5). In the broad-neck cultivars the increase in differential growth ratio in the neck region from stages I + II to stage III was much smaller (30.8 ± 12.4%). There was no change in the differential growth ratio in the stylar region of the broad-neck cultivars throughout development (Fig. 5). The regression equations for the individual cultivars of the two categories of fruit are summarized in Supplemental Tables S1 and S2 for the neck and the stylar regions, respectively.

**Fig. 6:**
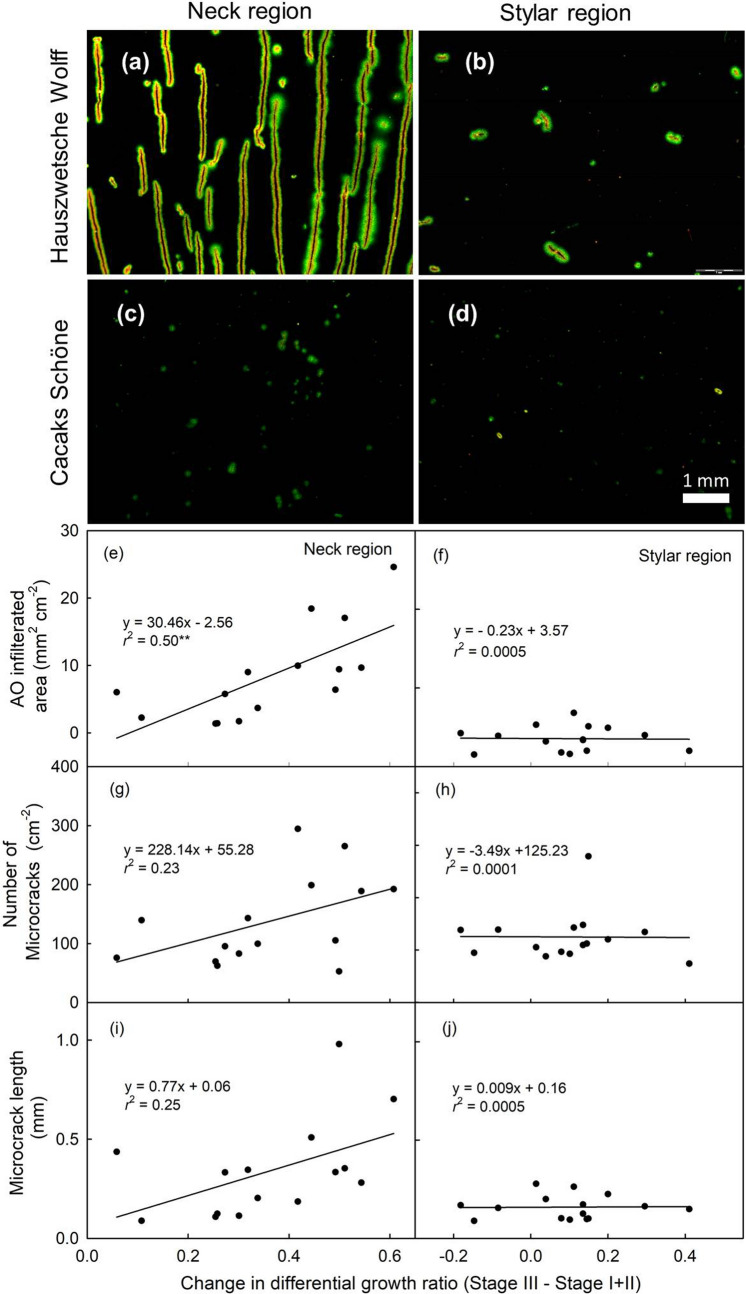
Fluorescence microscope images of the neck (**a**, **c**) and stylar (**b**, **d**) regions of the fruit of European plums cvs. ‘Hauszwetsche Wolff’ (**a**, **b**) and ‘Cacaks Schöne’ (**c**, **d**). Relationship between acridine orange (AO) infiltrated area (**e**, **f**) or the number of microcracks per unit area (**g**, **h**) or the microcrack length (**i**, **j**) and the increase in constant differential growth ratio in the neck region (**e**, **g**, **i**) and stylar region (**f**, **h**, **j**) of various plum cultivars. The cultivars used comprised both broad-neck and narrow-neck cultivars

Microscopic inspection revealed extensive cuticular microcracking in the neck region of the shrivel-susceptible cv. ‘Hauszwetsche Wolff’, but only a little microcracking in the stylar region. There was essentially no microcracking in either region of the shrivel non-susceptible cv. ‘Cacaks Schöne’ (Fig. 6a–d). In both cultivars the center region was intermediate between neck and stylar region. When the data were pooled for the narrow- and broad-neck cultivars, a significant positive relationship was obtained between microcracking in the neck region, as indexed by the area infiltrated by the fluorescent tracer AO and the difference in differential growth ratios between stage III and stage I + II (Fig. 6e). There were no such differences for the stylar region (Fig. 6f).

The increase in microcracking in the neck region was due to increases in both the microcrack number and microcrack length (Fig. 6g, i). There were no such changes in the stylar region (Fig. 6 h, j).

**Fig. 7:**
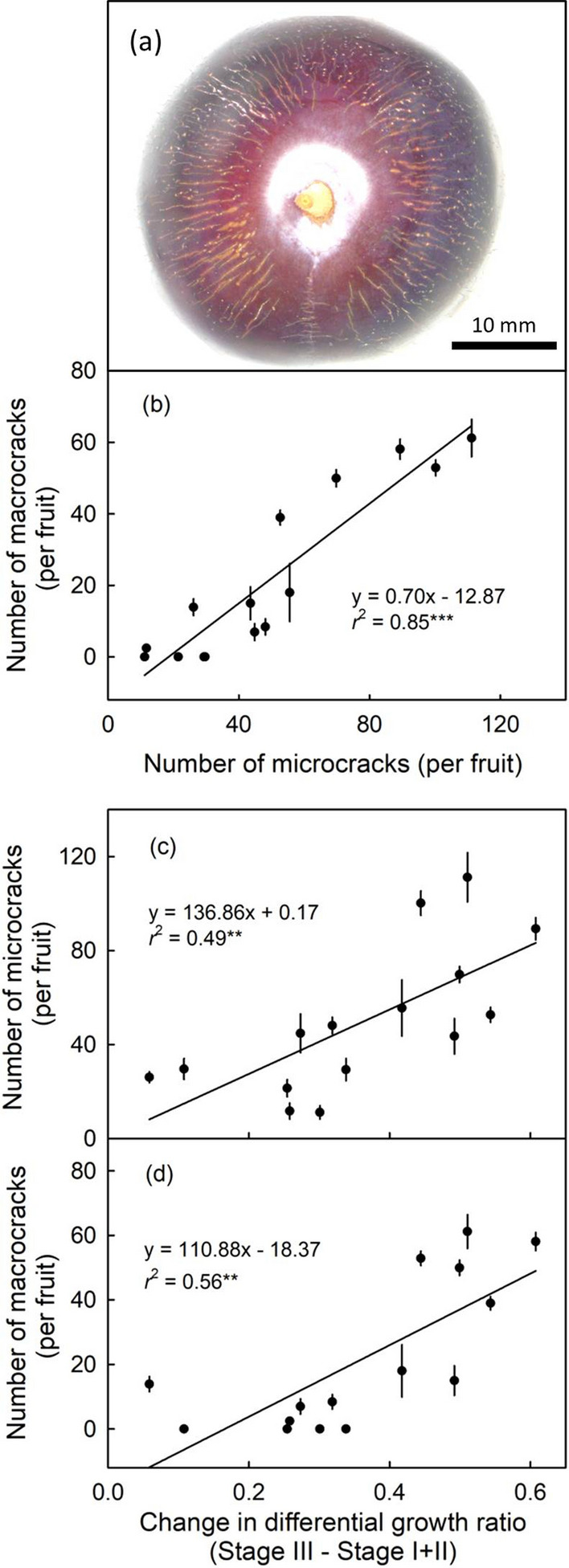
**a** Mature plum in top view showing numerous radially oriented macroscopically visible macrocracks in the fruit skin. **b** Relationship between the number of macrocracks and of microcracks in the neck region of the fruit of selected plum cultivars. The cultivars used comprised broad-neck and narrow-neck cultivars. **c**, **d** Relationship between the number of the microcracks (**c**) or macrocracks (**d**) per fruit in the neck region and change in surface expansion rate in the neck region of the fruit of various plum cultivars

Fruit of the narrow-neck cultivars often exhibited macroscopically-visible, radially-oriented ‘macrocracks’ in the neck region, orientated towards the pedicel – like bicycle-wheel spokes to the hub (Fig. 7a). The frequency of macrocracking in this region was a linear function of the number of microcracks (Fig. 7b). Both, the numbers of micro- and macro-cracks per fruit, were positively and significantly related to the difference in the constant differential growth ratio between stage III and stage I + II (Fig. 7c and d).

## Discussion

Our results indicate that, in the narrow-neck cultivars, the sharp increase in the constant differential growth ratio in the neck region during stage III, gives rise to particularly high rates of lateral growth strain, these give rise to high rates of radial microcracking, and these to high rates of radial macrocracking, and these to neck shrivel. As background, the deposition of cutin ceases and microcracking increases during stage III (Knoche and Peschel 2007). For this reason, the sudden increase in constant differential growth ratio in the neck region of the narrow-neck cultivars, must suddenly distribute a roughly constant amount of cutin over a rapidly-increasing area of fruit surface (Knoche and Lang 2017). Thus the cutin redistribution occurs at a markedly higher rate in the neck region, than in the stylar region. Supplementary support for this hypothesis comes from the highly-orientated cuticular microcracks, reported both here and in an earlier study (Knoche et al. 2019). These microcracks are orientated radially – like the spokes in a bicycle wheel, towards the hub. In the narrow-neck cultivars, the stage III filling-in of the flesh volume in the neck region causes lateral growth strain resulting in this typical pattern of cuticle failure. The close relationship between radial cuticular microcracking and radial skin macrocracking, orientation indicates that the macrocracks arise from the microcracks.

There are no such microcracks in the stylar region of the shrivel-susceptible cultivars because this filling-in took place earlier, during stage I + II, when cutin deposition was still ongoing. That wax synthesis and deposition continue throughout stage III does not contradict the above, because wax is deposited as monomers and, hence, has little effect on the fracture properties of the cuticle (Khanal and Knoche 2017).

The above arguments also account for the absence of micro- and macro-cracking in the shrivel non-susceptible, broad-neck cultivars. Here, the growth pattern is more uniform (allometric) and the critical filling-in of the neck and stylar regions takes place earlier when cutin deposition is still ongoing. The newly-added cutin during this earlier phase, is likely able to ‘fix’ the cuticular strain as the surface gradually expands. Such a behavior has already been shown for apples (Khanal et al. 2013, 2014).

At present we do not know the physiological basis of the late filling-in in some plum cultivars. For other stone fruit crops, stage I development occurs primarily by cell division whereas stage III growth is primarily by cell expansion (for cherries see Lilleland and Newsome 1934; Tukey and Young 1939; for olives see Rapoport et al. 2004). There is no info for European plum. Thus, it is not known whether differences in the pattern of cell division and/or cell expansion account for the differential growth habit of broad-neck and narrow-neck plum cultivars. Also, the reason for the cessation of cuticle deposition after stage I + II development in plum is not known (Knoche and Peschel 2007). European plum and sweet cherry may also be similar in this respect. In sweet cherry, cuticle deposition ceases during stage II development due to a downregulation of genes involved in cuticle synthesis and deposition (Peschel et al. 2007; Alkio et al. 2012). In both species, the cessation of cuticle deposition approximately coincided with the onset of color change. Fatty acids (C16 and C18 fatty acids) are precursors of cutin monomers and wax components and their synthesis takes place in the stroma of plastids (Li-Beisson et al. 2013; Li et al. 2016). Thus, it may be speculated that a dismantling of chloroplasts during color change is contributing to the cessation of cuticle deposition in both fruit crops.

## Practical implications

From a practical point of view the question arises whether neck shrivel can be avoided under field conditions. The answer is probably no. Neck shrivel cannot be avoided by cultural means since the late filling of the neck appears to be a genotypic character of each cultivar. Clearly, environmental factors that affect the growth rate on the one hand and the rate of cuticle deposition on the other hand will affect the delicate balance between surface expansion and strain fixation by cuticle deposition. The higher the rate of surface expansion relative to the rate of strain fixation, the larger will be the likelihood for neck shrivel. From a breeder’s perspective, selecting broad-neck genotypes and eliminating narrow-neck genotypes will decrease the likelihood of neck shrivel.

### *Author contribution statement*

 MK and BPK conceived and designed research. MK obtained the funds to support the study. BPK and MK planned the experiments. BPK, AB, and DA conducted the experiments. BPK, AB, DA and MK analysed the data. BPK and MK wrote, revised, and edited the manuscript. All authors read and approved the manuscript.

## Supplementary Information

Below is the link to the electronic supplementary material.

Supplementary file1 (DOCX 202 KB)

## Data Availability

The datasets generated during and/or analysed during the current study are available from the corresponding author on reasonable request.
