# Supplementary material for: Neck shrivel in European plum is caused by cuticular microcracks, resulting from rapid lateral expansion of the neck late in development
Source: Planta. 2023 Aug 5;258(3):62. doi: 10.1007/s00425-023-04218-9 (PMC10404172; doi:10.1007/s00425-023-04218-9)
Supplement: Supplementary file 1 — Supplementary file1 (DOCX 202 KB) [file 425_2023_4218_MOESM1_ESM.docx]

**Neck shrivel in European plum is caused by cuticular microcracks, resulting from rapid lateral expansion of the neck late in development**

Journal name: Planta

Bishnu P. Khanal, Anil Bhattarai, Divya Aryal, Moritz Knoche

Institute for Horticultural Production Systems, Leibniz University Hannover, Herrenhäuser Straße 2, 30419 Hannover, Germany

Corresponding author

Moritz Knoche

Email: moritz.knoche@obst.uni-hannover.de

Supplementary data file

| **Table S1**. Estimates of regression parameter of the relationships of log transformed diameters (in mm) in the neck region with log transformed center diameter (in mm) of the fruit of various European plum cultivars. | | | | | | |
| --- | --- | --- | --- | --- | --- | --- |
|  | Stage I+II | | | Stage III | | |
| Cultivars | Intercept ± SE | Slope ± SE | *r*^2^ | Intercept ± SE | Slope ± SE | *r*^2^ |
| **Narrow neck cultivars** |  |  |  |  |  |  |
| Auerbacher | 0.26 ± 0.05 | 0.62 ± 0.04 | 0.88*** | - 0.37 ± 0.05 | 1.12 ± 0.04 | 0.92*** |
| Doppelte Hauszwetsche | 0.05 ± 0.03 | 0.47 ± 0.02 | 0.96 | 0.20 ± 0.05 | 1.01 ± 0.04 | 0.89*** |
| Hauszwetsche Gunser | 0.31 ± 0.05 | 0.56 ± 0.04 | 0.84*** | - 0.41 ± 0.05 | 1.17 ± 0.03 | 0.93*** |
| Hauszwetsche Wolff | 0.24 ± 0.03 | 0.62 ± 0.02 | 0.94*** | - 0.30 ± 0.04 | 1.07 ± 0.03 | 0.93*** |
| Jofella | 0.27 ± 0.03 | 0.60 ± 0.03 | 0.93* | - 0.41 ± 0.07 | 1.11 ± 0.05 | 0.87*** |
| Topper | 0.32 ± 0.04 | 0.59 ± 0.04 | 0.88*** | - 0.30 ± 0.05 | 1.08 ± 0.03 | 0.93*** |
| Tulare | 0.09 ± 0.04 | 0.79 ± 0.03 | 0.94* | - 0.31 ± 0.07 | 1.09 ± 0.05 | 0.89*** |
| **Mean** | **0.22 ± 0.04** | **0.61 ± 0.04** |  | **-0.33 ± 0.03** | **1.09 ± 0.02** |  |
| **Broad neck cultivars** |  |  |  |  |  |  |
| Cacaks Schöne | - 0.07 ± 0.03 | 0.99 ± 0.02 | 0.98* | - 0.26 ± 0.05 | 1.10 ± 0.03 | 0.95*** |
| Chrudiminer | 0.09 ± 0.04 | 0.81 ± 0.04 | 0.94* | - 0.48 ± 0.06 | 1.23 ± 0.04 | 0.91*** |
| Czernowitzer | - 0.10 ± 0.04 | 0.96 ± 0.03 | 0.96* | - 0.52 ± 0.06 | 1.24 ± 0.04 | 0.95*** |
| Elena | 0.04 ± 0.02 | 0.87 ± 0.02 | 0.99 | - 0.06 ± 0.05 | 0.93 ± 0.03 | 0.9 |
| Juna | - 0.11 ± 0.05 | 0.99 ± 0.04 | 0.95* | - 0.48 ± 0.07 | 1.24 ± 0.05 | 0.92*** |
| Lützelsacher | - 0.02 ± 0.05 | 0.91 ± 0.04 | 0.95 | - 0.48 ± 0.08 | 1.23 ± 0.06 | 0.9*** |
| Stanley | 0.12 ± 0.03 | 0.80 ± 0.03 | 0.97*** | - 0.24 ± 0.04 | 1.05 ± 0.03 | 0.95*** |
| Vandor | 0.09 ± 0.05 | 0.83 ± 0.04 | 0.95 | - 0.42 ± 0.07 | 1.17 ± 0.04 | 0.93*** |
| **Mean** | **0.004 ± 0.03** | **0.90 ± 0.03** |  | **-0.37 ± 0.06** | **1.15 ± 0.04** |  |
| The slopes of the relationships were significant at *P* ≤ 0.001 (***), 0.01 (***) or 0.05 (*). The minimum number of fruit per cultivar and per sampling time point was 12. | | | | | | |

| **Table S2**. Estimates of regression parameters of the relationships of log transformed diameters (in mm) in the stylar region with log transformed center diameter (in mm) of the fruit of various European plum cultivars. | | | | | | |
| --- | --- | --- | --- | --- | --- | --- |
|  | Stage I+II | | | Stage III | | |
| Cultivars | Intercept ± SE | Slope ± SE | *r*^2^ | Intercept ± SE | Slope ± SE | *r*^2^ |
| **Narrow neck cultivars** |  |  |  |  |  |  |
| Auerbacher | - 0.01 ± 0.04 | 0.84 ± 0.04 | 0.95 | - 0.01 ± 0.05 | 0.86 ± 0.04 | 0.88 |
| Doppelte Hauszwetsche | 0.88 ± 0.03 | 0.41 ± 0.02 | 0.21*** | 0.04 ± 0.04 | 0.82 ± 0.03 | 0.88 |
| Hauszwetsche Gunser | 0.12 ± 0.04 | 0.72 ± 0.04 | 0.93** | - 0.24 ± 0.05 | 1.02 ± 0.04 | 0.89*** |
| Hauszwetsche Wolff | 0.05 ± 0.03 | 0.79 ± 0.02 | 0.97 | - 0.19 ± 0.04 | 0.99 ± 0.03 | 0.92*** |
| Jofella | - 0.04 ± 0.03 | 0.91 ± 0.02 | 0.98 | - 0.21 ± 0.04 | 1.02 ± 0.02 | 0.96*** |
| Topper | 0.00 ± 0.04 | 0.87 ± 0.03 | 0.95 | - 0.16 ± 0.04 | 1.00 ± 0.03 | 0.95*** |
| Tulare | 0.14 ± 0.04 | 0.74 ± 0.03 | 0.94** | - 0.07 ± 0.07 | 0.89 ± 0.05 | 0.83 |
| **Mean** | **0.16 ± 0.12** | **0.75 ± 0.06** |  | **-0.12 ± 0.04** | **0.94 ± 0.03** |  |
| **Broad neck cultivars** |  |  |  |  |  |  |
| Cacaks Schöne | - 0.20 ± 0.04 | 1.07 ± 0.03 | 0.98*** | - 0.04 ± 0.05 | 0.92 ± 0.03 | 0.92 |
| Chrudiminer | - 0.18 ± 0.04 | 1.03 ± 0.03 | 0.97*** | - 0.12 ± 0.04 | 0.94 ± 0.03 | 0.94** |
| Czernowitzer | - 0.17 ± 0.04 | 1.03 ± 0.03 | 0.97*** | - 0.28 ± 0.07 | 1.07 ± 0.04 | 0.91*** |
| Elena | - 0.10 ± 0.03 | 0.96 ± 0.03 | 0.98** | - 0.26 ± 0.05 | 1.06 ± 0.04 | 0.91*** |
| Juna | - 0.20 ± 0.04 | 1.06 ± 0.04 | 0.97*** | 0.02 ± 0.06 | 0.88 ± 0.04 | 0.88 |
| Lützelsacher | - 0.09 ± 0.04 | 0.96 ± 0.03 | 0.97* | - 0.32 ± 0.08 | 1.10 ± 0.06 | 0.88*** |
| Stanley | - 0.06 ± 0.04 | 0.92 ± 0.03 | 0.97 | - 0.19 ± 0.04 | 1.00 ± 0.03 | 0.93*** |
| Vandor | 0.07 ± 0.03 | 0.84 ± 0.03 | 0.97* | - 0.15 ± 0.07 | 0.99 ± 0.04 | 0.90* |
| **Mean** | **-0.12 ± 0.03** | **0.98 ± 0.03** |  | **-0.16 ± 0.04** | **1.00 ± 0.03** |  |
| The slopes of the relationships were significant at *P* ≤ 0.001 (***), 0.01 (***) or 0.05 (*). The minimum number of fruit per cultivar and per sampling time point was 12. | | | | | | |


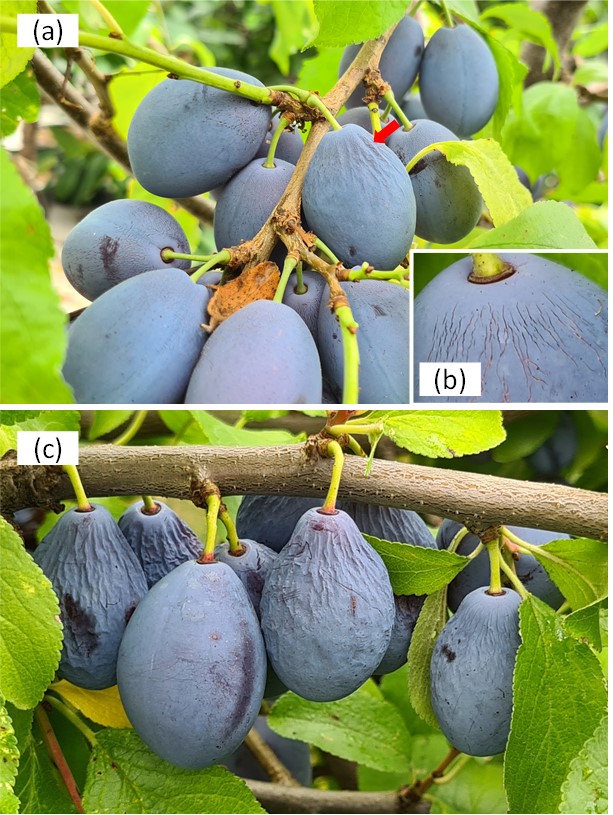


**Fig. S1**. Fruit of ‘Hauszwetsche Wolff’ with and without symptoms of the neck shrivel. **a** Single neck shriveled fruit (marked with red arrow) in a group of non-shriveled fruit. **b** Detail of neck region of symptomatic fruit showing macrocracks. **c** Single non-shriveled fruit in a group of severely shriveled fruit.

**Fig. S2** **a** Relationship between calculated and measured lengths of the microcracks. **b** Relationship between measured microcrack lengths and area per microcrack of the fruit of different European plum cultivars.
